# Ghrelin Amplifies the Nicotine-Induced Release of Dopamine in the Bed Nucleus of Stria Terminalis (BNST)

**DOI:** 10.3390/biomedicines11092456

**Published:** 2023-09-04

**Authors:** Jázmin Ayman, Miklós Palotai, Roberta Dochnal, Zsolt Bagosi

**Affiliations:** 1 Department of Obstetrics and Gynecology, Albert Szent-Györgyi School of Medicine, University of Szeged, H-6701 Szeged, Hungary; aymanjazmin@gmail.com; 2 Department of Radiology, Brigham and Women’s Hospital, Harvard Medical School, Boston, MA 02115, USA; palotai@bwh.harvard.edu; 3 Department of Pediatrics and Pediatric Health Center, Albert Szent-Györgyi School of Medicine, University of Szeged, H-6701 Szeged, Hungary; rdochnal@gmail.com; 4 Department of Pathophysiology, Albert Szent-Györgyi School of Medicine, University of Szeged, H-6725 Szeged, Hungary

**Keywords:** ghrelin, nicotine, dopamine, BSNT, superfusion

## Abstract

Ghrelin is an orexigenic neuropeptide that is known for stimulating the release of growth hormone (GH) and appetite. In addition, ghrelin has been implicated in addiction to drugs such as nicotine. Nicotine is the principal psychoactive component in tobacco and is responsible for the reward sensation produced by smoking. In our previous in vitro superfusion studies, it was demonstrated that ghrelin and nicotine stimulate equally the dopamine release in the rat amygdala, and ghrelin amplifies the nicotine-induced dopamine release in the rat striatum. However, less attention was paid to the actions of ghrelin and nicotine in the bed nucleus of the stria terminalis (BNST). Therefore, in the present study, nicotine and ghrelin were superfused to the BNST of male Wistar rats, and the dopamine release from the BNST was measured in vitro. In order to determine which receptors mediate these effects, mecamylamine, a non-selective nicotinic acetylcholine receptor (nAchR) antagonist, and GHRP-6, a selective growth hormone secretagogue receptor (GHS-R1A) antagonist, were also superfused to the rat BNST. Nicotine significantly increased the release of dopamine, and this effect was significantly inhibited by mecamylamine. Ghrelin increased dopamine release even more significantly than nicotine did, and this effect was significantly inhibited by GHRP-6. Moreover, when administered together, ghrelin significantly amplified the nicotine-induced release of dopamine in the BNST, and this additive effect was reversed partly by mecamylamine and partly by GHRP-6. Therefore, the present study provides a new base of evidence for the involvement of ghrelin in dopamine signaling implicated in nicotine addiction.

## 1. Introduction

Nicotine is the principal psychoactive component in tobacco that is responsible for the reward sensation produced by smoking [1]. Nicotine exerts its psychoactive effects through nicotinic acetylcholine receptors (nAchRs) expressed in the central nervous system (CNS) [2]. During smoking, nicotine gets into the blood circulation, passes the blood-brain barrier, reaches the brain within seconds, and activates the nAchR found in the midbrain, resulting in the release of dopamine that mediates the reward sensation produced by smoking [3,4]. Actually, there are three major dopaminergic pathways in the brain, called the mesolimbic, the nigrostriatal, and the tuberoinfundibular pathways [5]. The mesolimbic pathway emerges from the ventral tegmental area (VTA) and sends projections to the amygdala and the ventral striatum, the latter being represented by the nucleus accumbens (NAcc) but also the prefrontal cortex [6]. The mesolimbic pathway mediates reward sensation and affects and regulates cognitive functions such as learning and memory [2]. The nigrostriatal pathway arises from the substantia nigra pars compacta (SNc) and projects to the dorsal striatum, including the putamen and the nucleus caudatus (PNC) [7]. The nigrostriatal pathway modulates posture and motor behavior and mediates the learning of motor habits and decision-making [7,8]. The tuberoinfundibular pathway starts in the nucleus arcuatus (NArc), also known as the infundibular nucleus, located in the tuberal region of the hypothalamus, innervates the median eminence, and ends at the level of the anterior pituitary [9,10]. The dopamine released from these nerve endings tonically inhibits the secretion of prolactin in the anterior pituitary [9,10]. Nicotine stimulates the mesolimbic and nigrostriatal dopaminergic pathways via different subtypes of nAchR, resulting in the release of dopamine in the ventral striatum that mediates the sensation of reward but also the release of dopamine in the dorsal striatum that promotes locomotion [11,12]. This observation is supported by in vitro studies in which nicotine superfused to the striatum stimulated dopamine release in rats [13,14], and this stimulatory effect was prevented by superfusion of the non-selective nAchR antagonist mecamylamine [15,16]. The observation is also supported by in vivo studies, since nicotine infused into the striatum increased dopamine output and locomotor activity in freely moving rats, and these increasing effects were reversed by infusion of mecamylamine [15,16].

Ghrelin is an orexigenic neuropeptide that is known for stimulating the release of growth hormone (GH) and appetite [17,18]. Isolated first from the rat stomach, ghrelin can be transported through the blood and vagal nerve to exert its effects on the brain via the growth hormone secretagogue receptor (GHS-R). GHS-R has two isoforms, GHS-R1A and GHS-R1B [19]: GHS-R1A plays a role in the regulation of food intake and energy balance, but also drug addiction [20], while GHS-R1B seems to modulate the activity of GHS-R1A [21]. There is a growing body of evidence demonstrating that ghrelin plays an important role in the reward sensation produced by addictive drugs such as alcohol, amphetamine, cocaine, morphine, and nicotine [4,22,23,24,25,26]. Ghrelin contributes to the reward sensation, probably acting through the GHS-R1A scattered along the so-called cholinergic-dopaminergic reward link [27,28,29,30,31,32]. The cholinergic-dopaminergic reward link consists of the afferent cholinergic projection that starts in the laterodorsal tegmental area (LDTA) and projects to the VTA, and the mesolimbic, mesocortical, or mesolimbicocortical dopaminergic pathway that emerges from the VTA and sends projections to the amygdala, the ventral striatum represented by the NAcc, and the prefrontal cortex [33]. This is demonstrated by previous in vitro studies in which ghrelin superfused to the striatum and the amygdala and increased dopamine release in both brain regions [34,35]. This is also demonstrated by in vivo studies in which ghrelin injected peripherally or directly into the VTA increased dopamine release in the NAcc, increasing locomotor activity and food consumption in rats [36,37,38]. 

**Figure 1:**
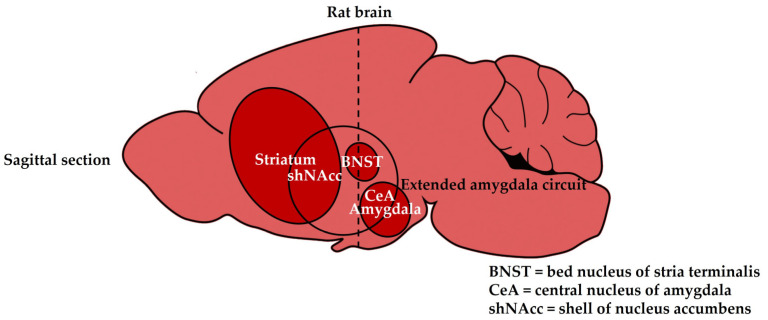
Sagittal section of the rat brain. The BNST is an important relay station in the extended amygdala circuit that consists of the central nucleus of the amygdala (CeA), the bed nucleus of the stria terminalis (BNST), and the shell of the nucleus accumbens (shNAcc).

As regards the interaction between ghrelin and nicotine, previous studies focused mainly on their actions on the typical dopaminergic areas, such as the VTA, SNc, striatum, and amygdala, in which ghrelin and nicotine had a similar stimulatory effect on the extracellular dopamine output [22,27,28,29,30,31,32,34,35]. For example, in our previous in vitro superfusion studies, it was demonstrated that ghrelin and nicotine stimulate equally the dopamine release in the rat amygdala, and ghrelin amplifies the nicotine-induced dopamine release in the rat striatum [34,35]. However, less attention was paid to the actions of ghrelin and nicotine in the bed nucleus of the stria terminalis (BNST) [39,40,41,42]. The BNST was first described a century ago as a band or ridge of gray matter lying medially to the caudate nucleus [42]. Anatomically, it is located ventral to the septum, above and below the anterior commissure, and before the hypothalamus, and it is enclosed by the globus pallidus of the basal ganglia on both sides. The BNST can be divided into anterior and posterior divisions that are comprised of up to 18 subregions [43]. The anterior division includes the anterolateral, the anteromedial, the oval, the fusiform, the juxtacapsular, the rhomboid, the dorsomedial, the ventral, and the magnocellular nuclei [43]. The posterior division of the BNST comprises the principal, the interfascicular, and the transverse nuclei. Despite its small size in anatomical preparations, the BNST was postulated to have a key role in physiological and pathological processes, including stress, anxiety, and depression [43]. Functionally, the BNST is an important relay station in the extended amygdala circuit that consists of the central nucleus of the amygdala (CeA), the BNST, and the shell of the NAcc (shNAcc); the extended amygdala, including the BNST, is considered to be an interface between reward and stress systems, in which dopamine signaling could be implicated [44] (Figure 1).

Therefore, in the present study, nicotine and ghrelin were superfused to the BNST of male Wistar rats, and the dopamine release from the BNST was measured in vitro. In order to determine which receptors mediate these effects, mecamylamine, a non-selective nAchR antagonist, and GHRP-6, a selective GHS-R1A antagonist, were also superfused to the rat BNST.

## 2. Materials and Methods

### 2.1. Animals 

Male Wistar rats weighing 150–250 g (N = 6) were used for each in vitro experiment. The rats were treated in accordance with the ARRIVE guidelines, and the experiments were carried out in accordance with the EU Directive 2010/63/EU for animal experiments. They were housed together and kept in their home cages at a constant temperature on a standard illumination schedule with 12 h light and 12 h dark periods (lights on at 6:00). Commercial food and tap water were available ad libitum. After all, the rats were decapitated; however, every effort was made to limit the number of animals used and minimize animal suffering. 

### 2.2. Substances 

The agonists used, such as nicotine and ghrelin, were provided by B. Braun Inc., Melsungen, Germany, and Bachem Inc., Bubendorf, Switzerland, respectively. The antagonists used, such as mecamylamine and GHRP-6, were purchased from Sigma-Aldrich Inc., St. Louis, MO, USA. The Krebs solution contained 113 mM NaCl, 4.7 mM KCl, 1.2 mM MgSO_4_, 25 mM NaHCO_3_, 11.5 mM glucose, 1.2 mM KH_2_PO_4_, and 2.5 mM CaCl_2_, solutions that were provided by Reanal Ltd., Budapest, Hungary. The tritium-labeled dopamine ([3H]DA) and the Ultima Gold scintillation fluid were ordered from Perkin-Elmer Inc., Waltham, MA, USA. The superfusion system itself was purchased from MDE Ltd., Heidelberg, Germany, and the method itself was originally described by Gaddum [45].

### 2.3. Dissection

After the rats were decapitated, their brains were rapidly removed and dissected in a Petri dish filled with an ice-cold Krebs solution. The BNST was isolated and extracted according to a stereotaxic atlas of the rat brain [46]. The stereotaxic coordinates were antero-posterior = +7.8 mm from the interaural line; lateral = 4 mm from the medial suture; and dorso-ventral = −5.8 mm from the skull. After extraction, the BNST was cut into slices of 300 μM with a McIlwain tissue chopper. 

### 2.4. Incubation 

The BNST slices obtained were incubated in 8 mL of Krebs solution, submerged in a water bath at 37 °C, and gassed through a single-use needle with a mixture of 5% CO_2_ and 95% O_2_ for 30 min. During the incubation, 5 μL of [3H]DA with a concentration of 1 mCi/mL and a specific activity of 60 Ci/mmol was added to the incubation medium with a Hamilton microliter syringe. 

### 2.5. Superfusion 

Six BNST slices were transferred to each of the cylindrical chambers of the superfusion system and superfused with Krebs solution for 30 min. A multichannel peristaltic pump (Gilson Minipuls 2, Gilson, Middleton, WI, USA) was used to maintain the superfusion rate constant at 300 μL/min. After 30 min of superfusion, the BNST slices were superfused for 32 min more, and during this time the fractions were collected in Eppendorf tubes by a multichannel fraction collector (Gilson FC 203B, Gilson, Middleton, WI, USA). 

### 2.6. Treatment 

The BNST slices were treated with 100 μM nicotine and/or 1 μM ghrelin 20 min after the superfusion had started and eventually pre-treated with 100 μM mecamylamine and/or with 1 μM GHRP-6, a selective GHS-R1A antagonist, 10 min after the superfusion had started. The doses of the agonists (nicotine and ghrelin) and antagonists (mecamylamine and GHRP-6) were based on previous in vitro superfusion studies [34,35]. 

### 2.7. Electrical Stimulation 

After 32 min of superfusion (that means that after 2 min of the time, the fractions were collected in Eppendorf tubes), electrical stimulation was carried out. The electrical stimulation consisted of square wave impulses with a voltage of 100 V, a pulse length of 5 ms, and a frequency of 10 Hz. The electrical impulses were delivered to each of the four chambers for 2 min, as golden electrodes were previously attached to both halves of the superfusion chambers and connected to an ST-02 electrical stimulator. 

### 2.8. Homogenization 

After the next 30 min of superfusion, the remaining BNST slices were removed and solubilized in 200 mL of Krebs solution using an ultrasonic homogenizer (Branson Sonifier 250, Emerson, St. Louis, MO, USA). The homogenized slices and the collected samples were mixed with 3 mL of Ultima Gold scintillation fluid in glass vials. 

### 2.9. Measurement 

The glass vials were transferred to a liquid scintillation spectrometer (Tri-carb 2100TR, Packard, Conroe, TX, USA) that measured the radioactivity emitted from the vials. The fractional [3H]DA release was calculated as a percentage of the radioactivity present in the collected samples relative to that of the remaining slices, which were expressed in counts per minute (CPM). 

### 2.10. Statistical Analysis 

Statistical analysis of the results was performed by repeated measures of analysis of variance followed by Tukey’s post-hoc test for pair-wise comparison (Stata13, StataCorp, College Station, TX, USA). A probability level of 0.05 or less (*p* ≤ 0.05) was accepted as indicating a statistically significant difference. 

## 3. Results

**Figure 2:**
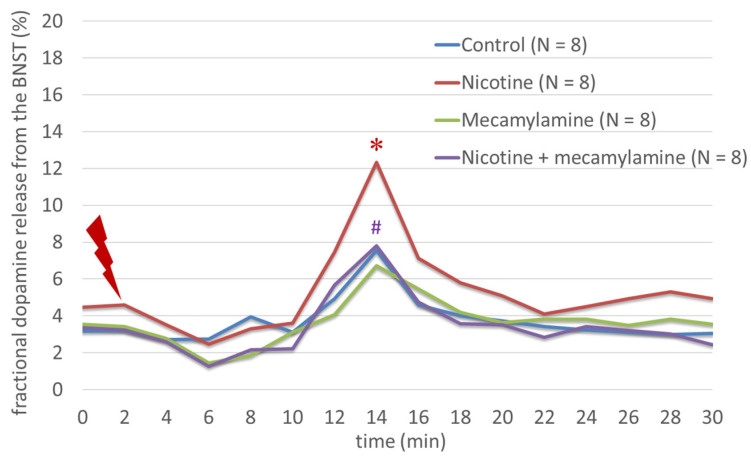
The effects of nicotine and mecamylamine on the dopamine release from the BNST. Nicotine significantly increased the fractional [3H]DA release from rat BNST after electrical stimulation, an effect that was significantly inhibited by mecamylamine. * indicates a statistically significant difference for agonist vs. control, whereas # indicates a statistically significant difference for agonist + antagonist vs. agonist alone.

**Figure 3:**
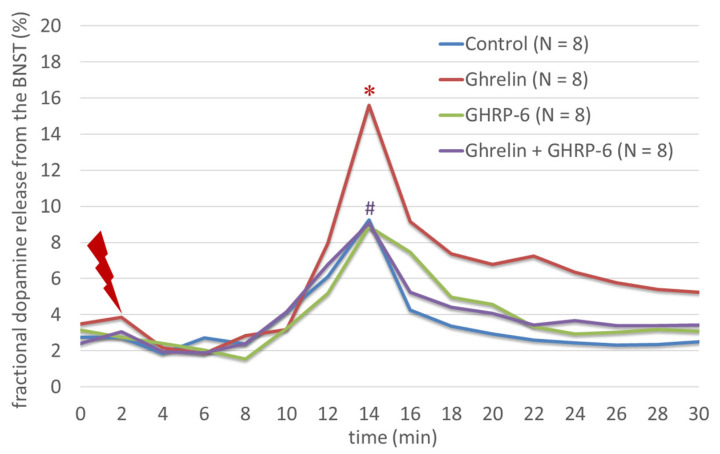
The effects of ghrelin and GHRP-6 on the dopamine release from the BNST. Ghrelin significantly increased the fractional [3H]DA release from rat BNST after electrical stimulation, an effect that was significantly inhibited by GHRP-6. * indicates a statistically significant difference for agonist vs. control, whereas # indicates a statistically significant difference for agonist + antagonist vs. agonist alone.

Nicotine significantly increased the fractional [3H]DA release from rat BNST after electrical stimulation (F(3,31) = 19.78; *p* < 0.001), an effect that was inhibited significantly by mecamylamine (F(3,31) = 19.78; *p* < 0.001) (Figure 2). Ghrelin increased even more significantly the fractional [3H]DA release from rat BNST after electrical stimulation than nicotine did (F(3,31) = 16.58; *p* < 0.001), an effect that was inhibited significantly by GHRP-6 (F(3,31) = 16.58; *p* < 0.001) (Figure 3).

**Figure 4:**
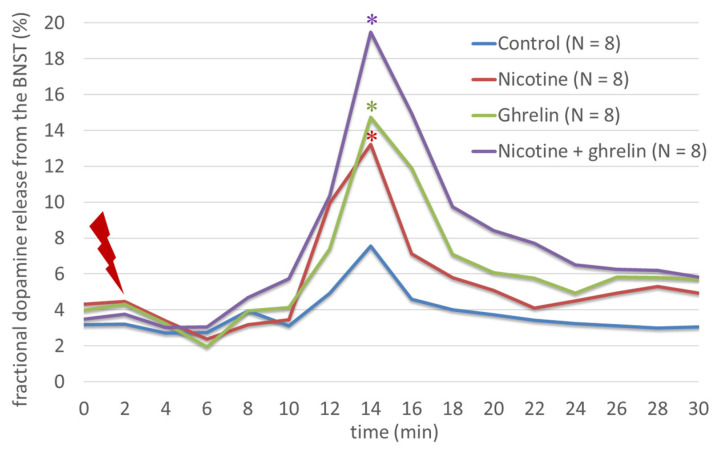
The effects of nicotine and ghrelin on the dopamine release from the BNST. Ghrelin significantly amplified significantly the nicotine-induced fractional [3H]DA release from rat BSNT after electrical stimulation. * indicates a statistically significant difference for agonist vs. control.

**Figure 5:**
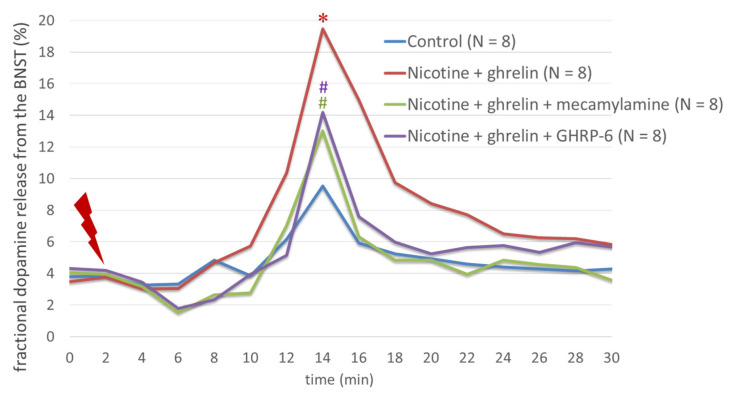
The effects of nicotine, ghrelin, mecamylamine, and GHRP-6 on the dopamine release from the BNST. The additive effect of nicotine and ghrelin on the fractional [3H]DA release from rat BSNT after electrical stimulation was partly reversed by mecamylamine and partly by GHRP-6. * indicates a statistically significant difference for agonist vs. control, whereas # indicates a statistically significant difference for agonists + antagonists vs. agonists.

Moreover, when administered together, ghrelin significantly amplified the nicotine-induced release of fractional [3H]DA release from rat BSNT after electrical stimulation (F(3,31) = 13.19; *p* < 0.001) (Figure 4), and this additive effect was partly reversed by mecamylamine (F(3,31) = 16.58; *p* < 0.001) and GHRP-6 (F(3,31) = 13.5; *p* < 0.05) (Figure 5).

## 4. Discussion

Nicotine significantly increased the release of dopamine from the rat BNST after electrical stimulation, and this effect was inhibited significantly by mecamylamine, a non-selective nAchR antagonist. This finding is concordant with previous in vivo and in vitro studies, which indicated that nicotine stimulates dopamine release in several subcortical and cortical brain regions [47,48,49,50]. This stimulatory effect of nicotine on dopamine must be mediated by different nAchR subtypes expressed on the dopaminergic terminals found in the BNST [1] and may contribute to the reward sensation produced by nicotine [5,51]. As we mentioned before, the BNST is a heterogeneous brain region that can be divided into anterior and posterior divisions and that can be further divided into 18 subregions [43]. Both the anterior division (that includes the anterolateral, the anteromedial, the oval, the fusiform, the juxtacapsular, the rhomboid, the dorsomedial, the ventral, and the magnocellular nuclei) and the posterior division (that comprises the principal, the interfascicular, and the transverse nuclei) receive and send distinct cholinergic, noradrenergic, dopaminergic, GABA-ergic, and glutamatergic projections [43]. Dopaminergic inputs that may underlie the rewarding action of nicotine originate from the VTA and periaqueductal gray (PAG), project primarily into the dorsolateral subdivision of the BNST, and synapse directly onto CRF neurons [52]. However, which of the two major dopaminergic inputs (originating in the VTA or PAG) to the BNST drives this behavior is not known [52]. There are multiple studies demonstrating that dopamine signaling in the BNST is implicated in the sensation of reward produced by addictive drugs, such as nicotine. Dose-dependent increases of extracellular dopamine in the BNST were observed after the administration of artificial drugs such as nicotine [53]. Moreover, increased release of dopamine in the BNST was described following exposure to natural rewarding substances, such as sucrose [54,55]. Furthermore, blocking dopamine (D1) receptors in the BNST reduced ethanol and sucrose self-administration [56]. Nevertheless, there are several studies suggesting that dopamine signaling in the BNST may also play a role in the negative effects induced by nicotine withdrawal [57,58]. In accordance, neurons within diverse nuclei and subnuclei of the BNST were demonstrated to co-express a variety of neuropeptides, such as corticotropin-releasing factor (CRF) and neuropeptide Y [59], and the dysfunction of these neuropeptides may contribute to stress reactions, anxiety, and depression, which can be observed during nicotine withdrawal [43].

Ghrelin increased even more significantly the release of dopamine from the rat BNST after electrical stimulation than nicotine did, and this effect was inhibited significantly by GHRP-6, a selective GHS-R1A antagonist. This finding is supported by in vivo studies, which reported that the administration of ghrelin induces the concomitant release of ventral tegmental acetylcholine and accumbal dopamine, which is associated with the locomotor hyperactivity induced by ghrelin [22,29,30,32]. The stimulatory effect of ghrelin on dopamine and locomotion must be mediated by GHS-R1A scattered along the cholinergic-dopaminergic reward link, which consists of the afferent cholinergic projection that starts in the LDTA and projects to the VTA, and the mesocortical or mesolimbicocortical dopaminergic pathway that emerges from the VTA and, among others, sends projections to the ventral striatum represented by the NAcc [27,28,60,61,62]. Activation of the cholinergic-dopaminergic reward link by ghrelin may lead to the stimulation of dopamine release from the dopaminergic terminals found in the BNST [1] and may induce a similar reward sensation to that produced by nicotine [5,50]. One of the most important dopamine inputs to the BNST arises from the VTA and projects into the dorsolateral subdivision of the BNST [57]. However, most of the BNST neurons are GABA-ergic (~97%) and some of them are glutamatergic (~3%) [43]. Therefore, ghrelin may activate locally the GABA-ergic and glutamatergic neurons, which may have an impact on the dopaminergic terminations within the BNST [54,55,56,60,63]. Generally, changes in the firing rate of dopaminergic, GABA-ergic, and glutamatergic neurons and axons in the BNST can evoke distinct affective states and motivated behaviors that may participate in both positive and negative reinforcement of drugs, including nicotine [64,65]. Continuously seeking the positive reinforcement produced by nicotine and avoiding the negative reinforcement induced by nicotine withdrawal can result in a relapse to smoking, especially in periods of stress, and spiral into nicotine addiction [1,65]. Specifically, the GABA-ergic and glutamatergic inputs of the anterior BNST may emerge from several cortical and limbic brain regions, such as the prefrontal cortex and the basolateral nucleus of the amygdala (BLA), innervate different nuclei of the BNST, such as the oval nucleus, and project to other limbic regions, such as the VTA and CeA [44]. The posterior BNST receives GABA-ergic input from the lateral septum, different nuclei of the amygdala, and glutamatergic input from the paraventricular region of the thalamus and different regions of the hippocampus. It innervates the dorsolateral subdivision of the BNST and sends projections to the ventral striatum, dorsal striatum, and paraventricular nucleus (PVN) of the hypothalamus, through which it modulates the activity of the hypothalamic-pituitary-adrenal (HPA) axis, also known as the stress axis [43]. Ghrelin is believed to activate the HPA axis directly by increasing the release of CRF at the hypothalamic level; however, an interaction of ghrelin with CRF at the level of the BNST cannot be excluded either [17,37]. In addition, the anterior division of the BNST is rich in CRF receptor type 1 (CRFR1), whereas the posterior division is richer in CRF receptor type 2 (CRFR2) [43], and a dysbalance between these receptors may contribute to hyperactivity of the HPA axis, anxiety, and depression, which can be observed during nicotine withdrawal [57]. 

**Figure 6:**
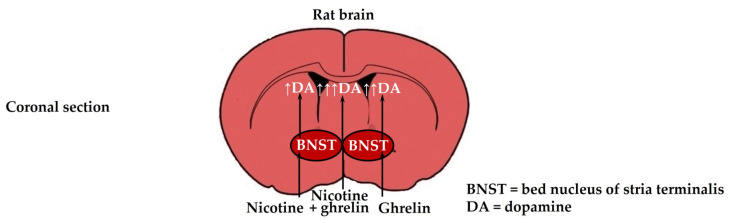
Coronal section of the rat brain. Nicotine increased significantly the release of dopamine (DA) from the rat bed nucleus of stria terminalis (BNST), ghrelin increased it even more significantly than nicotine did, and, when administered together, ghrelin amplified significantly the nicotine-induced release of dopamine from the rat BNST.

Moreover, when administered together, ghrelin significantly amplified the nicotine-induced release of dopamine from the rat BNST after electrical stimulation, and this effect was partly reversed by mecamylamine and partly by GHRP-6 (Figure 6).

Our previous in vitro superfusion studies led to similar results, as ghrelin and nicotine stimulated equally the dopamine release in the rat amygdala, and ghrelin amplified even more the nicotine-induced dopamine release in the rat striatum [30,31]. Despite the different anatomical aspects and physiological functions attributed to the BNST, amygdala, and striatum, there is a strong functional correlation between these brain regions. On the one hand, the BNST is an important relay station for both the cholinergic-dopaminergic reward link and the extended amygdala circuit. On the other hand, the dopaminergic neurons of the VTA send projections to each part of the extended amygdala, including the shNAcc, the BNST, and the CeA [10]. Thereby, besides the amygdala and the striatum, a possible place of interaction between ghrelin and nicotine could be the BNST, and the mechanism would be the activation of cholinergic, GABA-ergic, or glutamatergic neurons with an impact on the dopaminergic terminations found at the level of the BNST. Both animal experiments and human studies indicate the existence of an interaction between ghrelin and addictive drugs such as nicotine [25,66]. Most notably, ghrelin was implicated in reward sensation and drug-seeking behavior induced by alcohol, and the ghrelin receptor was suggested to be a possible pharmacological target in the treatment of alcohol addiction [67,68,69,70,71,72,73,74,75,76]. Nevertheless, there is also an evident connection between ghrelin and nicotine. For instance, higher plasma ghrelin levels were found in smokers when compared to non-smokers [77,78]. However, the level of salivary ghrelin was found to decrease acutely after smoking a cigarette, and plasma ghrelin also decreased after a certain period of abstinence from tobacco, which was considered a rebound effect after smoking [79,80,81]. Some authors even suggested that ghrelin levels could be used as a biomarker for increased risk of relapse to smoking [82,83,84,85]. Consequently, they revealed that ghrelin levels have a prognostic value that is independent of the severity of the negative effects characteristic of nicotine withdrawal or craving [82,83,84,85]. Overall, these studies indicate a positive association between smoking and ghrelin levels, an association that is reversed during abstinence, particularly among those likely to remain abstinent. Based on the previous and present studies, ghrelin may be used as a prognostic tool, and the ghrelin receptor may serve as a pharmacological target in the treatment of nicotine addiction. 

## 5. Conclusions

The present study provides a new base of evidence for the contribution of ghrelin to dopamine signaling implicated in nicotine addiction. In addition, the amygdala and the striatum, a possible place of interaction between ghrelin and nicotine could be the BNST, and the mechanism would be the activation of cholinergic, GABA-ergic, or glutamatergic neurons with an impact on the dopaminergic terminations found at the level of the BNST. However, the intimate mechanism of how ghrelin interacts with nicotine remains to be elucidated. Both animal experiments and human studies indicate the existence of an interaction between ghrelin and addictive drugs, such as nicotine. Taken together, these studies indicate a positive association between smoking and ghrelin level, an association that is reversed during abstinence, particularly among those likely to remain abstinent. Therefore, our preclinical study may have clinical implications, as it suggests that ghrelin may be used as a prognostic tool and the ghrelin receptor may serve as a pharmacological target in the treatment of nicotine addiction.

## Data Availability

The datasets generated during the current study are available from the corresponding author upon reasonable request.
